# Association of Systemic Sclerosis and Periodontitis with Vitamin D Levels

**DOI:** 10.3390/nu13020705

**Published:** 2021-02-23

**Authors:** Gaetano Isola, Giuseppe Palazzo, Alessandro Polizzi, Paolo Murabito, Clemente Giuffrida, Alberto Lo Gullo

**Affiliations:** 1 Department of General Surgery and Surgical-Medical Specialties, School of Dentistry, University of Catania, 95124 Catania, Italy; gpalazzo@unict.it (G.P.); alexpoli345@gmail.com (A.P.); paolo.murabito@unict.it (P.M.); 2 IRCCS Centro Neurolesi “Bonino Pulejo”, 98124 Messina, Italy; clemente.giuffrida@irccsme.it (C.G.); albertologullo@virgilio.it (A.L.G.)

**Keywords:** vitamin D, diet, systemic sclerosis, periodontal disease, C-reactive protein, clinical trial

## Abstract

The aim of the present study was to analyze the association among systemic sclerosis (SSc), periodontitis (PT); we also evaluated the impact of PT and SSc on vitamin D levels. Moreover, we tested the association with potential confounders. A total of 38 patients with SSc, 40 subjects with PT, 41 subjects with both PT and SSc, and 41 healthy controls were included in the study. The median vitamin D levels in PT subject were 19.1 (17.6–26.8) ng/mL, while SSc + PT group had vitamin d levels of 15.9 (14.7–16.9) ng/mL, significantly lower with respect to SSc patients (21.1 (15.4–22.9) ng/mL) and to healthy subjects (30.5 (28.8–32.3) ng/mL) (*p* < 0.001). In all subjects, vitamin D was negatively associated with c-reactive protein (CRP) (*p* < 0.001) and with probing depth (PD), clinical attachment level (CAL), bleeding on probing (BOP), and plaque score (PI) (*p* < 0.001 for all parameters) and positively related to the number of teeth (*p* < 0.001). Moreover, univariate regression analysis demonstrated an association among high low-density lipoproteins (LDL) cholesterol (*p* = 0.021), CRP (*p* = 0.014), and PT (*p* < 0.001) and reduced levels of vitamin D. The multivariate regression analysis showed that PT (*p* = 0.011) and CRP (*p* = 0.031) were both predictors of vitamin D levels. Subjects with PT and SSc plus PT had significant lower vitamin D values with respect to SSc and to healthy subjects. In addition, PT seems negatively associated with levels of vitamin D in all analyzed patients.

## 1. Introduction

Systemic sclerosis (SSc) is an autoimmune connective disease characterized by imbalance regulation of the immune system and micro and macrovascular deformities, which can lead to excessive deposition of collagen, resulting in skin and internal organ fibrosis [1,2]. During the last few decades, the pathogenesis of SSs has been focused on microvasculopathy and endothelial dysfunction, principally manifested by digital ulcers and Raynaud’s phenomenon, which could determine progressive tissue fibrosis and, finally, organ tissue damages [3]. Recently, different studies have demonstrated, in SSc, an association between endothelial dysfunction, cardiovascular disease (CVD), and atherosclerosis [4,5].

Periodontitis (PT) is a chronic disease of the periodontal tissue due to different periodontal pathogens that induce an inflammatory response that could determine destruction of periodontium, alveolar bone resorption and, finally, loss of the tooth [6]. PT has been correlated with a plethora of systemic disorders, including endothelial dysfunction [7], diabetes [8], metabolic syndrome [9], and SSc [10].

Because both SSc and PT share many common factors, such as inflammation and vascular dysfunction [11], patients with PT and with SSc may have an increased risk of developing inflammation. For these reasons, it is of interest to find biomarkers, which could help better understand the etiology of SSc and prevent risk-related factors associated with SSc.

Vitamin D is a fat-soluble vitamin gained from an endogenous release from the skin subsequent to sunlight exposure or from food [12,13]. Several studies have described the role of vitamin D as a regulator during inflammation phases in chronic inflammatory diseases [14,15,16]. Growing evidence has suggested that increased vitamin D has a positive effect on both systemic and oral health. In particular, it seems that vitamin D acts as an immunomodulator through adaptive and innate immune responses; in addition, vitamin D could have a key role in the endothelium and vessels homeostasis to maintain vascular health in chronic conditions, including SSc [17,18].

At the same time, some studies have evidenced that lower vitamin D levels can determine an augmented risk of periodontal inflammation [19] that could lead to significant tooth loss [20]. In this regard, it has also been demonstrated that, during PT, vitamin D, through its specific receptor expressed on the surface of cells of the immune system, has a protective effect at the endothelial level of the periodontal tissue; this, in turn, causes a marked release of the B and T lymphocytes that are usually produced as a result of the tissue invasion of periodontal pathogens [21].

However, studies on vitamin D and gingival tissues resulted in few preliminary and contradictory results [22,23]. Recently, a trial, which evaluated the effect of serum vitamin D in patients with PT, described a direct association between low vitamin D and active phases of PT [22]. Conversely, a similar cohort from the National Health and Nutrition Examination Survey (NHANES) III study demonstrated a 10% odds reduction of bleeding on probing for every 30 nmol/L rises of vitamin D in serum, even if it was not able to demonstrate a statistical correlation between vitamin D and PT [23].

In accordance with this evidence, this study aimed to analyze the association of SSc and PT and vitamin D levels and the significant trend of this association. Moreover, in the analyzed sample, it was explored whether vitamin D levels were influenced by some possible predictors. Additionally, we tested if PT had a significant influence on vitamin D levels.

## 2. Materials and Methods

### 2.1. Study Design

From January 2013 to May 2017, 274 subjects were firstly enrolled at the School of Dentistry (PT patients and healthy controls) and at the Unit of Rheumatology (SSc patients and healthy controls) of the University of Messina, Messina, Italy. The University of Messina International Review Board (IRB) approved the study protocol. All enrolled patients gave written informed consent, in which all study risks and characteristics were specified. The study was performed in accordance with the Declaration of Helsinki, revised in 2000. The study followed the Strengthening the Reporting of Observational Studies in Epidemiology (STROBE) guidelines (Supplementary Materials Table S1). The study was registered on clinicaltrials.gov (NCT04066842).

Patients were selected by sex and within a specific age range (from 40 to 65 years old), in order to obtain a similar proportion of cases in the categories—sex and age—defined by the selection variable. A total of 49% of patients were males, with an age range from 46 to 59 years.

In this initial stage, by the same calibrated clinician, patient characteristics were recorded, such as age, sex, body mass index (BMI), and a full medical anamnesis, including all drugs taken. BMI was valued by calculating the patient weight and height. Moreover, each patient underwent a full oral examination that comprised a periodontal charting at six sites per tooth [24] by a manual probe (UNC-15, Hu-Friedy, Chicago, IL, USA).

The limited and diffuse SSc disease subsets were classified according to the American College of Rheumatology classification criteria [25]. The SSc onset was diagnosed by the first non-Raynaud symptom derivable to the SSc [26]. The diagnosis of PT was performed, as previously described [24], by (1) having a number of teeth ≥16; (2) bleeding on probing (BOP) in the least 40% of sites, a clinical attachment level (CAL) ≥2 mm, and a probing depth (PD) ≥4 mm [6]; (3) having ≥2 mm of interdental bone loss confirmed using RINN Rx (Dentsply Sirona Italia, Rome, Italy) [27].

The subjects enrolled as healthy patients did not present any pathologies, did not take drugs, had good oral health conditions, and did not present sites with PD ≥ 4 mm or CAL ≥ 4 mm, or signs of alveolar bone loss verified through periapical RINN X-rays.

For all enrolled patients, the exclusion criteria were: (1) consumption of contraceptive drugs; (2) pregnancy or lactation; (3) drinking; (4) consumption of any medications that could cause hyperplasia at the gingival tissues.

### 2.2. Population

**Figure 1:**
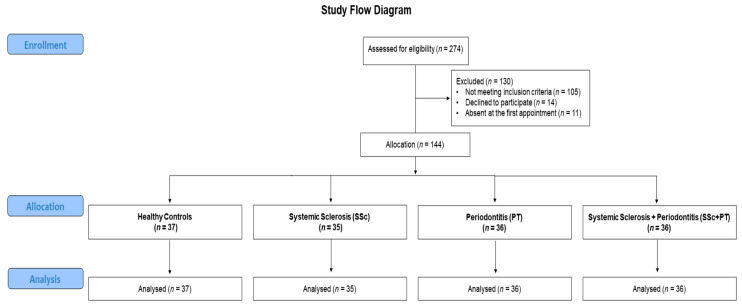
Workflow diagram of the study.

Following a first screening, 130 patients were excluded because they did not meet study inclusion criteria (*n* = 105), declined the study participation (*n* = 14), or were absent at the clinical examination (*n* = 11). After this phase, a final number of 35 patients with SSc, 36 patients with PT, 36 patients with both SSc and PT, and 37 healthy subjects were enrolled (Figure 1).

### 2.3. Data Collection

Vitamin D intake, reported in international units (IU), was assessed by a self-administered food frequency questionnaire (FFQ) authenticated for vitamin D intake evaluation [28,29]. The FFQ used in the study presented specific domains designed at determining the average common food intake and aimed at identifying the average vitamin and multivitamin supplementation daily intake.

Moreover, it evaluated the glycemic status and the presence of diabetes, which was calculated by the presence of a fasting blood glucose ≥7 mmol/L.

In SSc patients, possible signs and symptoms, such as joint tenderness with synovitis, calcinosis, telangiectasia, contractures, and eventual organ involvement (e.g., gastrointestinal tract or lungs), were documented. The appearance of enlarged capillaries, pericapillary hemorrhages at the nail fold, and capillary dropout, such as early signs of probable major organ involvement were recorded. In SSc patients, a single calibrated examiner evaluated and quantified the modified Rodnan skin score (MRSS). As previously demonstrated, MRSS assessed the cutaneous involvement of SSc (from grade 0, normal skin to grade 3, intense skin thickness) at 51 points in over 17 regions of the body [30].

The periodontal evaluation included the recording of PD, CAL, and plaque score (PI) [31]. BOP was recorded by the appearance of gingival bleeding after probing up to 30 s during PD assessment. CAL was documented, having the cementoenamel junction as a reference, as PD, plus the presence of gingival recession. All indices were recorded at six sites per present tooth.

The performed inter-examiner reliability, analyzed using the intraclass correlation coefficient (ICC) resulted in a good reliability agreement for CAL (ICC = 0.821), which was the primary outcome chosen. The intra-examiner reliability was performed for CAL on six random patients per group and resulted in a good degree of agreement for both principal (ICC = 0.807) and control (ICC = 0.816) examiners.

### 2.4. Laboratory Analyses

On each patient, at the first visit and in the same center, was performed a venous sampling at 8:00 a.m. Levels of fibrinogen, glucose, and plasma lipids were obtained by routinely laboratory analysis. The c-reactive protein (CRP), expressed in milligrams per deciliters (mg/dL), were obtained using an enzyme-linked immunoassay (ELISA) kit.

The vitamin D (25-OH vitamin D3) levels were obtained with a commercial ELISA kit, in accordance with the instructions of the manufacturer (Immunodiagnostics System, Boldons, United Kingdom) and were reported as nanograms per milliliter (ng/mL), in a measurement range of 9.6–66.4 ng/mL.

In each batch was included a masked duplicate sample of control plasma that had a variation coefficient within one pair of 4.7% for vitamin D and an inter-assay variation of 5.2% (for vitamin D). In order to reduce the bias regarding the seasonal variation due to the intensity of sunlight skin exposition, all patients were enrolled each year from March to July.

### 2.5. Power Sample Size

Because the study hypothesis was that PT and SSc patients had augmented CAL, before the study, the sample size was determined by considering 4 groups of patients, an effect size of 0.26 for the CAL, a two-sided significance level of 0.05, a standard deviation of 0.5 [32], and a power level of 80%. It was established that about 28 patients were needed per single group in order to achieve a good power sample. However, around 35 subjects per group were finally enrolled, obtaining a power value of 81%. The analysis was performed using statistical power analysis software (G*Power version 3.1.9.4, Universitat Dusseldorf, Dusseldorf, Germany).

### 2.6. Statistical Analysis

Numerical data were represented as mean ± SD or median and interquartile range, while categorical variables were represented using number and percentage. Because most of the examined variables did not present a normal distribution as demonstrated through the Kolmogorov–Smirnov test, a non-parametric approach was used.

To evaluate the numerical data in the four groups, the Kruskal–Wallis test was used and the Mann–Whitney test was applied for the comparison between two single groups. The Bonferroni correction was calculated by the comparison between groups with a significant α-level of 0.050; for the present analysis, the adjusted level of significance was 0.050/6 = 0.008.

The non-parametric Spearman test was used in order to evaluate significant correlations and interdependence among vitamin D and periodontal indices, in each single patient, and in all patients together. To better evaluate the possible association among diabetes, (expressed as yes/no) and vitamin D levels, a point-biserial correlation was used.

A stepwise multivariable linear regression analysis was applied to establish the dependence of each periodontal index, by such variables as gender, age, BMI, vitamin D levels, dietary- and supplementary vitamin D intake, CRP, total cholesterol, high-density lipoprotein (HDL)- and LDL cholesterol.

A possible presence of multicollinearity among variables and between vitamin D levels, dietary and supplemental vitamin D intake was explored and quantified by assessing the variance inflation factors (VIF). To evaluate if periodontal parameters increased or decreased with a vitamin D increase, *p*-trend quartiles of vitamin D were calculated, and for each vitamin D quartile, a mean and standard deviation (±SD) of each periodontal parameter was calculated, and the Jonckheere–Terpstra test was used to analyze the *p*-trend of vitamin D levels for each periodontal index.

Furthermore, uni- and multivariable linear regression models were applied to establish the dependence of vitamin D by some predictors, such as age, sex, education level, smoking (yes/no), triglycerides, LDL- and HDL cholesterol, and using possible confounders, such as BMI, CRP, SSc, and PT. *p*-value < 0.05 was set as significant. All analyses were made by a statistical software program (SPSS 22.0 for Windows package, SPS srl, Bologna, Italy).

## 3. Results

### 3.1. Patient Population

All subjects were matched for age (*p* = 0.057), sex (*p* = 0.131), BMI (*p* = 0.081), and number of smokers (*p* = 0.412) (Table 1).

Patients with SSc (0.4 (0.31–0.47) mg/dL), PT (0.58 (0.41–0.65) mg/dL), and SSc + PT (0.59 (0.39–0.67) mg/dL) had increased median levels of CRP when compared to healthy controls (0.31 (0.25–0.34) mg/dL) (*p* < 0.001 for all evaluations) (Table 1).

Regarding vitamin D levels, 14.2% (*n*= 5) of patients with SSc presented a deficiency of vitamin D in serum (≤20 ng/mL). A further comparison in median values of vitamin D evidenced that patients with PT (19.1 (17.6–26.8) ng/mL), and with SSc + PT group (15.9 (14.7–16.9) ng/mL) had significantly lower median vitamin D levels in comparison with SSc (21.1 (15.4–22.9) ng/mL) and healthy subjects (30.5 (28.8–32.3) ng/mL) (*p* < 0.001) (Table 1; Table 2). Moreover, the intake of dietary (*p* = 0.152) and supplementary (*p* = 0.812) vitamin D were similar among groups.

Regarding periodontal indices, PT and PT + SSc patients had a significantly lower number of teeth and augmented PD, CAL, and BOP levels in comparison to SSc and healthy controls (*p* < 0.001), while PI values did not differ among groups (*p* = 0.097) (Table 1).

### 3.2. Primary Outcome

**Figure 2:**
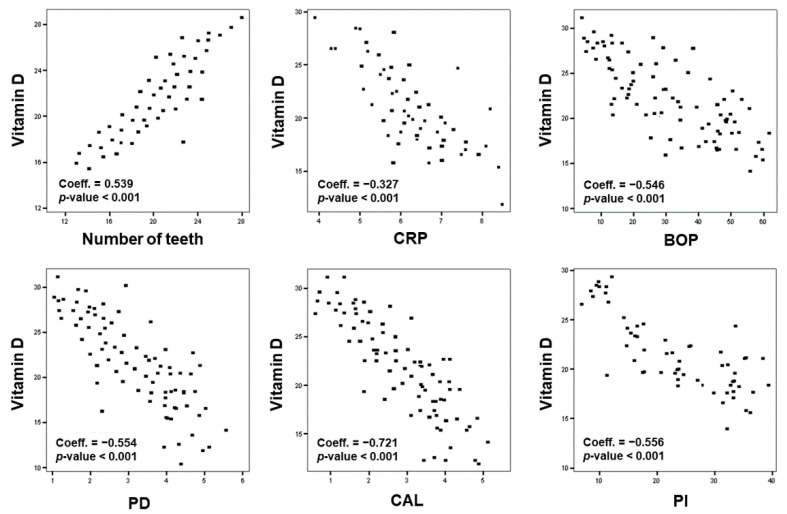
Correlation analysis among vitamin D levels, CRP, and periodontal parameters (Correlation Spearman’s). Coeff., coefficient.

The Spearman correlation analysis evidenced that serum vitamin D was positively correlated with the number of teeth (*p* < 0.001) and a negative correlation with high levels of CRP (*p* < 0.001), CAL (*p* < 0.001), PD (*p* < 0.001), BOP (*p* < 0.001), and PI (*p* < 0.001) (Figure 2).

The stepwise analysis evidenced that, in all enrolled patients, the number of teeth, PD, CAL, and BOP were correlated with serum vitamin D (*p* < 0.001 for all outcomes) (Table 3). In particular, the number of teeth was correlated with age (*p* = 0.021), female gender (*p* = 0.007), and CRP (*p* < 0.001); CAL was significantly correlated with age (*p* = 0.043), CRP (*p* < 0.001), and vitamin D (*p* < 0.001). PD was correlated with CRP (*p* < 0.001) and vitamin D (*p* < 0.001); BOP was correlated with age (*p* = 0.008), CRP (*p* < 0.001), and vitamin D (*p* < 0.001) (Table 3). The other analyzed confounders did not show any significant association for the analyzed outcomes.

There were no multicollinearities among vitamin D, dietary and supplemental vitamin D, with a VIF that resulted in 1.26 for vitamin D and dietary vitamin D; 1.39 for vitamin D and supplementary vitamin D; 1.51 for dietary and supplemental vitamin D.

**Figure 3:**
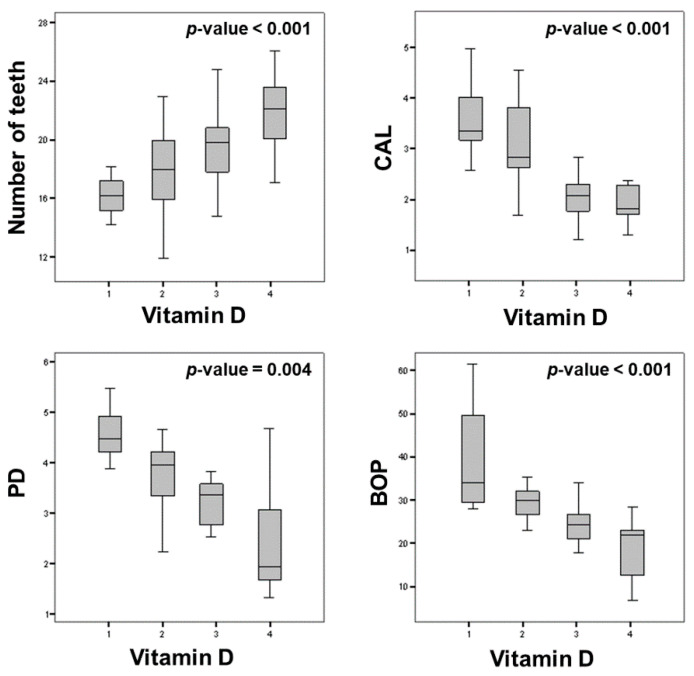
*p*-trend analysis of increase or decrease vitamin D levels for each single periodontal index.

The *p*-trend analysis evidenced that there was a significant progressive increase of serum vitamin D with a proportional increase in number of teeth (*p* < 0.001), while there was a proportionate decrease in serum vitamin D with a proportional increase in PD (*p* = 0.004), CAL (*p* = 0.001), and BOP (*p* < 0.001) (Figure 3).

Lastly, the univariate regression analysis evidenced that high LDL cholesterol (*p* = 0.021), CRP (*p* = 0.014), and PT (*p* < 0.001) were significant predictors of serum vitamin D.

The multivariate regression analysis evidenced that PT (*p* = 0.011) and CRP (*p* = 0.031) were significant predictors of serum vitamin D (Table 4).

## 4. Discussion

This trial was designed to analyze the correlation among serum vitamin D, SSc, and PT, and to identify the confounders, which had a significant influence on periodontal status in patients with SSc and PT. Moreover, the aims were to assess if PT had an influence on serum vitamin D.

The results have shown that patients with PT and SSc plus PT had significant low serum vitamin D levels compared to SSc and healthy subjects.

In accordance with the results of the present study, previous reports have been shown that patients with PT and with SSc presented serum vitamin D significantly lower than controls [33,34].

In this regard, some factors, such as the thickness of the skin, intestinal malabsorption, insufficient diet, and reduced sunlight exposure, may represent a key role of reduced vitamin D levels in SSc patients [35].

The major issue in the appearance of vascular related-disease during SSc was demonstrated to be the tissue hypoxia. Tissue hypoxia is one of the main causes for oxidative stress and relative vasospasm, which in turn leads to an imbalance of the coagulation system and endothelial injury, damage of vascular endothelial cells, and, finally, tissue fibrosis [36]. Recent evidence has been suggested concerning the antioxidative and anticoagulant effects of vitamin D, with the intriguing theory that vitamin D deficiency may accelerate vascular disorders in SSc through the stimulation of the oxidative stress pathways [37].

Moreover, recent studies supported that reduced levels of vitamin D could also have a role in the pathogenesis of SSc through a modulation action of vitamin D in the regulation of transforming growth factor (TGF)-β, a crucial mediator in the production of fibroblast and collagen during both SSc [38] and PT [39]. Consequently, vitamin D could interfere and cause the fibrosis of the skin in SSc, which may also cause temporomandibular joints and oral dysfunction, similar to rheumatoid patients [40].

Moreover, recent evidence introduced the hypothesis that lower vitamin D serum could lead to an increase of autoimmune and inflammatory disease risk, especially during SSc. Vitamin D has been demonstrated to have a direct influence on the immune system through its vitamin D receptor, which can modulate the pro-inflammatory cytokines, lymphocytes, and the overall host response [41].

During the last few decades, many shreds of evidence have demonstrated the protecting role of vitamin D in oral tissues, especially during different diseases, such as PT [23,42].

In this regard, long prospective studies carried out on a large scale in the adult population have shown that subjects who presented high levels of vitamin D had a 20% lower risk of developing PT and tooth loss [23]. Furthermore, another trial on healthy pregnant women showed an increased risk odds ratio (OR) of 2.1 of developing PT in those who had vitamin D over 30 ng/mL [43].

Moreover, the essential role of vitamin D in regulating calcium levels in the human body, especially in the bone and soft tissues, has been unanimously recognized [44].

It has been demonstrated that vitamin D has combined anti-inflammatory and antimicrobial activities. Vitamin D, in fact, has a direct effect on the adaptive immune system, stimulating the production of molecules, such as cathelicidins and defensins, from different mediators and inflammatory cells, such as monocytes, lymphocytes, and macrophages with direct pro-inflammatory effects on all tissues, including periodontal tissues [45].

Indeed, a strong anti-inflammatory action exerted by vitamin D has been demonstrated, in a direct dose-dependent way, in the mouth, especially against the main periodontal pathogenic bacteria [45]. Grenier et al. [46] have highlighted a role in vitamin D through a selective inhibition on *Porphyromonas gingivalis* (*P. gingivalis*) and a specific gene suppression action of the associated virulence factor, which in turn determine a lower bacterial load of the *P. gingivalis* on gingival biofilm, which would reduce the subsequent inflammatory response and evident tissue damage during PT. In this regard, our study has shown that the number of teeth and the levels of periodontal indexes were significantly dependent on serum vitamin D (*p* < 0.001 for all observations). These results were in agreement with previous observations, which highlighted a possible relationship between vitamin D and PT; in this regard, cohort studies have shown that an increase in the severity of PT was related to low levels of vitamin D in serum [47].

Therefore, basing on this evidence, our analysis was aimed at further analyzing the relationship among serum levels of vitamin D and PT and the effects exerted by PT and gingival health vitamin D levels in healthy subjects and in subjects with PT and SSc.

Our study demonstrated that, in the enrolled patients, a proportional increase (*p*-trend) in the number of teeth and a proportional decrease of PT (CAL, PD, and BOP) was associated with a proportional increased in vitamin D levels in serum. Moreover, interestingly, the multivariate regression analysis evidenced that PT and CRP were the significant predictors of serum vitamin D.

In accordance with our study, some authors [22] reported that high levels of BOP was found in subjects who presented low vitamin D serum (<50 nmol/L), and a net risk to develop PT of 33%, compared to those who presented higher vitamin D levels (>50 nmol/L). In accordance, other reports have highlighted that patients with optimal serum levels of vitamin D had good gingival health and low BOP and CAL levels, with reduced levels of interleukin-6 (IL-6), one of the main mediators of the phase of tissue destruction during PT [48]. On this aspect, Zhan et al. [49] showed that proportional increases of 25-μg/L of vitamin D in serum were related to a reduced presence of PT and tooth loss, strengthening the hypothesis that elevated serum levels of vitamin D may represent immune protection in healthy patients with PT.

This has been confirmed by some studies that have shown that supplemental diet therapy with vitamin D and calcium supplements had positive effects, in terms of PD and BOP reduction in PT patients undergoing maintenance therapy [50,51]. However, in the present study, all enrolled subjects had the same supplemental and dietary vitamin D values.

It is interesting to note that, in our study, the number of teeth and the main periodontal indices were significantly dependent on vitamin D levels in serum, and that they correlated to the serum CRP levels in the multivariate analysis, which also was a significant negative predictor of serum vitamin D.

In agreement, with the capability of vitamin D reducing the inflammatory response, interfering on the release on the CRP has been previously demonstrated [52,53]. On the other hand, the correlation among vitamin D, CRP, and PT may be due to the role performed by PT on inflammation, which could lead to an adjunctive systemic release of CRP [54]. In agreement, a recent report [55] has evidenced that CRP was strictly correlated with PT, even after adjusting for some potential confounders [55,56,57]. For these reasons, during SSc, PT can lead to a negative systemic inflammatory stimulus and a relative augmented risk of endothelial dysfunction and focal ischemic injuries that, associated with SSc, may determine, in the heart tissues, myocytes apoptosis and fibroblasts replacement, with a final, irreversible myocardial fibrosis [56].

However, our trial has certain limits, such as the design of the study that does not permit analysis of the longitudinal relationship among vitamin D, SSc, and PT.

In the last few decades, there has been an increase in the number of studies aimed at better establishing the role of vitamin D in human health. Research is still ongoing, which aims to find and develop new methods to help clinicians better improve the conditions to determine PT and tooth loss (especially in some chronic conditions, including SSc).

The results of the present study suggested that subjects with PT and SSc plus PT had low serum vitamin D compared to SSc and healthy subjects. A progressive increase of serum vitamin D was associated with good oral health conditions. Moreover, PT and high CRP were predictors of serum vitamin D. However, further studies with a prospective design and a larger sample are needed to better understand the impact of vitamin D during PT and SSc.

## Figures and Tables

**Table 1:** Clinical features of all enrolled patients and a comparison among them. *p*-value < 0.05, statistically significant. Blood values are represented, such as median and interquartile range (IQR) (1st; 3rd).

Clinical Features	Controls (*n* = 37)	SSc (*n* = 35)	PT (*n* = 36)	SSc + PT (*n*= 36)	*p-*Value
Male, *n* (%)	18 (42.8)	16 (38)	18 (39.4)	15 (39.4)	0.131
Age, mean ± SD	52.9 ± 3.2	53.2 ± 3.5	52.7 ± 2.8	52.5 ± 2.9	0.057
Education level					
Primary school, *n* (%)	12 (28.6)	13 (31)	16 (34.9)	14 (34.1)	0.589
High school, *n* (%)	15 (35.7)	14 (33.3)	11 (30.2)	12 (34.1)	0.563
College/university, *n* (%)	10 (35.7)	9 (35.7)	9 (34.9)	10 (31.7)	0.558
Ethnicity					
White, *n* (%)	37 (100)	35 (100)	36 (100)	36 (100)	0.999
BMI, kg/m^2^, mean± SD	24.2 ± 3.3	23.7 ± 3.2	24 ± 2.6	23.8 ± 3.1	0.081
Glucose, mg/dL, median (1st; 3rd)	102.1 (85.9–105)	105.1 (91–105.6)	106.3 (98–102.3)	107.1 (99–110.3)	0.061
Uric acid, mg/dL, median (1st; 3rd)	2.2 (1.8–2.9)	3.3 (2.7–3.9)	4.2 (3.2–4.6)	4.6 (3.5–5.2)	<0.001
Albumin, g/L, median (1st; 3rd)	37.1 (29.7–39.4)	38.9 (35.5–40.6)	39.9 (36.1–43.9)	40.2 (35.8–44.1)	0.258
Fibrinogen, mg/dL, median, (1st; 3rd)	294.3 (259–310.3)	299.2 (262.3–306)	294.9 (284.9–318)	311 (284.9–322.3)	0.496
Apolipoprotein A, mg/dL, median (1st; 3rd)	136.8 (112–154.1)	138.9 (115–145.1)	132 (119–141.3)	138.6 (122–159)	0.358
Total cholesterol, mg/dL, median (1st; 3rd)	179.2 (169–187.2)	185.1 (177–189)	195.8 (181–202.3)	191.9 (184–196)	0.758
HDL-cholesterol, mg/dL, median (1st; 3rd)	52.3 (45–56.3)	53.9 (48.1–58.4)	56.8 (52.2–60)	57.1 (45–54)	0.661
LDL-Cholesterol mg/dL, median (1st; 3rd)	117.8 (98–121.3)	122.1 (117–128)	127 (116.3–131)	123.4 (119–129)	0.547
BUN, mg/dL, median (1st; 3rd)	29.6 (25.1–33.2)	31.5 (28.4–32.4)	31.8 (28.9–33)	33.1 (28–35.1)	0.496
CRP, mg/dL, median (1st; 3rd)	0.31 (0.25–0.34)	0.4 (0.31–0.47)	0.58 (0.41–0.65)	0.59 (0.39–0.67)	<0.001
Systolic pressure, mm/hg, median (1st; 3rd)	118 (115–123.1)	123.6 (112–129)	128.7 (119–142)	125.6 (121–132)	<0.001
Diastolic pressure, mm/hg, median (1st; 3rd)	86.3 (79.9–88.6)	82.5 (80–88.7)	85.5 (81.9–95)	84.6 (82.6–87)	0.024
Ferritin, ng/mL, median (1st; 3rd)	80.3 (77.1–85.2)	89.9 (84.2–92)	95.7 (92–99.2)	95.9 (91–97.5)	0.059
Smoker, *n* (%)	6 (19)	5 (21.4)	6 (21)	7 (24.4)	0.412
Current, *n* (%)	7 (16.6)	5 (14.3)	4 (16.3)	5 (19.5)	0.636
Never, *n* (%)	30 (81)	30 (78.6)	30 (79)	29 (75.6)	0.501
Past, *n* (%)	1 (2.3)	-	2 (4.6)	2 (4.9)	0.698
Comorbidities					
Diabetes, *n* (%)	-	3 (7.1)	4 (14)	4 (9.7)	<0.001
Atrial fibrillation, *n* (%)	-	2 (4.7)	2 (16.3)	3 (7.3)	<0.001
Angina pectoris, *n* (%)	-	3 (7.1)	3 (39.5)	4 (9.7)	<0.001
Stroke, *n* (%)	-	-	3 (27.9)	2 (2.4)	<0.001
Heart failure, *n* (%)	-	1 (2.3)	2 (23.5)	1 (2.4)	<0.001
Drug treatment of CVD					
Antihypertensive, *n* (%)	-	4 (9.5)	3 (32.5)	6 (14.6)	<0.001
Statins, *n* (%)	-	3 (7.1)	5 (25.6)	4 (9.7)	<0.001
Low-dose aspirin, *n* (%)	-	2 (4.7)	3 (30.2)	2 (9.7)	<0.001
Beta-blockers, *n* (%)	-	3 (7.1)	2 (23.5)	3 (12.2)	<0.001
SSc type, *n* (%)					
Diffuse cutaneous SSc, *n* (%)	-	32 (76.2)	-	30 (73.2)	
Limited cutaneous SSc, *n* (%)	-	5 (11.9)	-	6 (14.6)	
Overlap syndrome, *n* (%)	-	2 (4.7)	-	3 (7.3)	
Sclerosis sine scleroderma, *n* (%)	-	2 (4.7)	-	1 (2.4)	
Undifferentiated SSc, *n* (%)	-	1 (2.3)	-	1 (2.4)	
Disease duration					
≤5years, *n* (%)	-	19 (45.2)	-	20 (48.9)	
>5 to 10 years, *n* (%)	-	12 (28.6)	-	11 (26.8)	
≥10 years, *n* (%)	-	11 (26.2)	-	10 (24.4)	
Total SSc disease duration, median (1st; 3rd)	-	7.3 (5.9–7.9)	-	7.6 (5.8–8.2)	
MRSS, Total score		17.5 (16.4–18.3)		18.9 (15.6–20.4)	
Vitamin D					
Serum vitamin D, ng/mL, median (1st; 3rd)	30.5 (28.8–32.3)	21.1 (15.4–22.9)	19.1 (17.6–26.8)	15.9 (14.7–16.9)	<0.001
Dietary vitamin D intake (IU/Day), median (1st; 3rd)	207.4 (199.9–212)	202.6 (189–210.5)	200.1 (181.6–215)	199.1 (178.5–211)	0.152
Supplementary vitamin D intake (IU/Day), median (1st; 3rd)	246 (241.3–259)	239.9 (231.4–256)	235.4 (231–245)	248.5 (241–259.2)	0.812
Total vitamin D intake (IU/Day), median (1st; 3rd)	446.9 (440.1–452)	441.5 (429–451)	439.6 (429.5–446)	447.3 (412.3–458)	0.654
**Periodontal Parameters**					
Number of teeth, *n*, median (1st; 3rd)	25.9 (22–26)	22.6 (19–24)	18.3 (18–23)	17.9 (16–19)	<0.001
CAL, mm, median (1st; 3rd)	1.4 (1.1–1.8)	2.78 (2.1–3)	4.28 (2–2.9)	4.59 (3.1–5)	<0.001
Sites with CAL 4–5 mm (%) median (1st; 3rd)	-	-	11.7 (9.9–15.8)	35.7 (30.2–41.1)	<0.001
Sites with CAL ≥ 6 mm (%), median (1st; 3rd)	-	-	14.4 (1.9–18.8)	18.3 (15.9–25)	<0.001
PD, mm, median (1st; 3rd)	1.48 (1.31–1.6)	2.91 (2.6–3.5)	4.41 (2.68–3.21)	4.88 (4.12–5.2)	<0.001
Sites with PD 4–5 mm (%), median (1st; 3rd)	-	-	44.3 (2.1–5.1)	48.9 (40.2–53)	<0.001
Sites with PD ≥ 6 mm (%), median (1st; 3rd)	-	-	21.3 (11.8–33.6)	23.6 (17.8–29)	<0.001
BOP, median % (1st; 3rd)	9.1 (8.5–9.1)	11.8 (8.9–29)	36.6 (10.6–18.6)	41.1 (38.1–44.5)	<0.001
Plaque Index, median (1st; 3rd)	0.82 (0.71–0.9)	0.81 (0.71–0.9)	1.02 (0.78–1.89)	1.11 (0.71–0.95)	0.097

SSc, systemic sclerosis; PT, periodontitis; BMI, body mass index; HDL, high-density lipoprotein; LDL, light-density lipoprotein; BUN, blood urea nitrogen; CRP, C-reactive protein; CVD, cardiovascular disease; MRSS, modified Rodnan skin score; CAL, clinical attachment level; PD, probing depth.

**Table 2:** *p*-value of two-by-two comparison between groups performed by the Mann–Whitney test. Adjusted α level = 0.008.

Clinical Variables	CNT vs. SSc	CNT vs. PT	CNT vs. SSc + PT	SSc vs. PT	SSc vs. SSc + PT	PT vs. SSc + PT
Age	0.899	0.071	0.658	0.124	0.313	0.312
BMI, kg/m^2^	0.165	0.312	0.133	0.289	0.229	0.201
Glucose, mg/dL	0.923	0.589	0.158	0.587	0.587	0.209
Uric acid, mg/dL	0.002	0.002	0.002	0.079	0.078	0.454
Albumin, g/L	0.289	0.312	0.089	0.874	0.769	0.339
Fibrinogen, mg/dL	0.451	0.287	0.366	0.329	0.323	0.551
Apolipoprotein A, mg/dL	0.555	0.877	0.387	0.327	0.542	0.178
Total Cholesterol, mg/dL	0.489	0.598	0.358	0.331	0.312	0.694
Triglycerides, mg/dL	0.323	0.311	0.441	0.239	0.341	0.311
BUN, mg/dL	0.554	0.462	0.439	0.698	0.491	0.369
CRP, mg/dL	<0.001	<0.001	<0.001	0.641	0.555	0.002
Systolic pressure, mm/Hg	0.478	<0.001	<0.001	<0.001	<0.001	0.374
Diastolic pressure, mm/Hg	0.605	<0.001	0.004	<0.001	<0.001	0.321
Ferritin, mcg/L	0.709	0.443	0.399	0.444	0.532	0.557
Serum Vitamin D, ng/mL	<0.001	<0.001	<0.001	0.021	0.019	0.009
Dietary Vitamin D intake (IU/Day)	0.741	0.784	0.789	0.874	0.789	0.447
Supplementary Vitamin D intake (IU/Day)	0.753	0.589	0.448	0.811	0.531	0.589
Total Vitamin D intake (IU/Day)	0.589	0.854	0.599	0.423	0.339	0.872
**Periodontal Parameters**						
Number of teeth, *n*	<0.001	<0.001	<0.001	<0.001	0.487	<0.001
CAL, mm	<0.001	<0.001	<0.001	<0.001	0.565	<0.001
% of sites with CAL 4–5 mm	<0.001	<0.001	<0.001	<0.001	0.445	<0.001
% of sites with CAL ≥ 6 mm	<0.001	<0.001	<0.001	<0.001	0.333	<0.001
PD, mm	<0.001	<0.001	<0.001	<0.001	0.589	<0.001
% of sites with PD 4–5 mm	<0.001	<0.001	<0.001	<0.001	0.611	<0.001
% of sites with PD ≥ 6 mm	<0.001	<0.001	<0.001	<0.001	0.331	<0.001
BOP, %	<0.001	<0.001	<0.001	<0.001	0.339	<0.001
Plaque Index	0.129	0.223	0.151	0.213	0.154	0.299

CNT, healthy controls; SSc, systemic sclerosis; PT, periodontitis; BUN, blood urea nitrogen; CRP, C-reactive protein; IU, international unit; CAL, clinical attachment level; PD, probing depth; BOP, bleeding on probing.

**Table 3:** Stepwise linear regression models established for the number of teeth and periodontal indices. Age, vitamin D, dietary vitamin D, CRP, and LDL cholesterol were included as a continuous variable. For gender, female served as reference.

Variable	Number of Teeth			CAL		
	Coefficient	95% CI	*p*-Value	Coefficient	95% CI	*p*-Value
Age	−0.13	−0.21; −0.03	0.021	0.07	0.01; 0.55	0.043
Sex	1.62	0.59; 2.29	0.007	-	-	n.s.
Serum vitamin D	0.29	0.17; 0.41	<0.001	−0.15	−0.21; −0.55	<0.001
Dietary vitamin D	−0.19	−0.32; 0.22	0.83	-	-	n.s.
CRP	−2.66	−3.35; −1.79	<0.001	−0.39	0.24; 0.63	<0.001
LDL Cholesterol	-	-	n.s.	0.65	0.01; 0.79	0.089
**Variable**	**PD**			**BOP**		
	**Coefficient**	**95%C.I.**	***p*-value**	**Coeffficient**	**95%C.I.**	***p*-value**
Age	0.05	−0.03; 0.88	0.058	0.72	0.14; 1.46	0.008
Serum vitamin D	−0.15	−0.13; −0.78	<0.001	−1.77	−2.27; −1.31	<0.001
CRP	−0.37	−0.21; 0.54	<0.001	−4.21	−3.38; 9.26	<0.001
LDL Cholesterol	−0.17	−0.01; 0.23	0.085	-	-	n.s.

n.s., not significant. *p* < 0.05, statistically significant.

**Table 4:** Uni- and multivariate linear regression analysis. Age was included as a continuous variable. For PT, controls served as reference. For gender, male served as reference.

Variable	Univariate Model			Multivariate Model		
	B	95% CI	*p*	B	95% CI	*p*
Age (years)	−0.118	−0.036;0.214	0.331	−0.315	−0.469; 0.113	0.314
Sex	0.211	−0.054;0.329	0.108	0.218	−0.365; 0.178	0.087
Education	−0.096	−0.108;0.364	0.067	−0.103	−0.196; 0.102	0.745
Smoking	−0.369	−0.412;0.521	0.105	−0.252	−0.308;0.412	0.258
Triglycerides	−0.210	−0.259;0.654	0.369	−0.087	−0.095;0.396	0.265
LDL Cholesterol	−0.395	0.409;0.458	0.021	−0.354	−0.419;0.415	0.089
HDL Cholesterol	0.289	0.054;0.487	0.094	0.258	−0.114;0.412	0.109
BMI	−0.275	−0.301;0.331	0.328	−0.119	−0.174;0.258	0.156
PT	−0.356	−0.386;0.588	<0.001	−0.389	−0.159;0.661	0.011
SSc	−0.235	−0.326; 0.317	0.359	−0.208	−0.352;0.247	0.257
CRP	−0.202	−0.229;0.701	0.014	−0.447	−0.056;0.578	0.031

HDL, high-density lipoprotein; LDL, light-density lipoprotein; BMI, body mass index; PT, periodontitis; SSc, systemic sclerosis; CRP, C-reactive protein.

## Data Availability

Data available on reasonable request from the authors.

## Supplementary Materials

The following are available online at https://www.mdpi.com/2072-6643/13/2/705/s1, Table S1: STROBE Checklist.

## References

[1] Bellando-Randone S., Matucci-Cerinic M. (2017). Very Early Systemic Sclerosis and Pre-systemic Sclerosis: Definition, Recognition, Clinical Relevance and Future Directions. Curr. Rheumatol. Rep..

[2] Gueiros L.A., France K., Posey R., Mays J.W., Carey B., Sollecito T.P., Setterfield J., Bin Woo S., Culton D., Payne A.S. (2019). World Workshop on Oral Medicine VII: Immunobiologics for salivary gland disease in Sjögren’s syndrome: A systematic review. Oral Dis..

[3] Matucci-Cerinic M., Kahaleh B., Wigley F.M. (2013). Review: Evidence That Systemic Sclerosis Is a Vascular Disease. Arthritis Rheum..

[4] Man A., Zhu Y., Zhang Y., Dubreuil M., Rho Y.H., Peloquin C., Simms R.W., Choi H.K. (2012). The risk of cardiovascular disease in systemic sclerosis: A population-based cohort study. Ann. Rheum. Dis..

[5] Meier C., Freiburghaus K., Bovet C., Schniering J., Allanore Y., Distler O., Nakas C., Maurer B. (2020). Serum metabolites as biomarkers in systemic sclerosis-associated interstitial lung disease. Sci. Rep..

[6] Tonetti M.S., Greenwell H., Kornman K.S. (2018). Staging and grading of periodontitis: Framework and proposal of a new classification and case definition. J. Periodontol..

[7] Isola G., Polizzi A., Alibrandi A., Williams R.C., Leonardi R. Independent impact of periodontitis and cardiovascular disease on elevated soluble urokinase-type plasminogen activator receptor (suPAR) levels. J. Periodontol..

[8] Zeidán-Chuliá F., Yilmaz D., Häkkinen L., Könönen E., De Oliveira B.-H.N., Güncü G., Uitto V.-J., Caglayan F., Gürsoy U.K. (2018). Matrix metalloproteinase-7 in periodontitis with type 2 diabetes mellitus. J. Periodontal Res..

[9] Kim O.S., Shin M.H., Kweon S.S., Lee Y.H., Kim O.J., Kim Y.J., Chung H.J. (2017). The severity of periodontitis and metabolic syndrome in Korean population: The Dong-gu study. J. Periodontal Res..

[10] Isola G., Williams R.C., Gullo A.L., Ramaglia L., Matarese M., Iorio-Siciliano V., Cosio C., Matarese G. (2017). Risk association between scleroderma disease characteristics, periodontitis, and tooth loss. Clin. Rheumatol..

[11] Da Silva G.S.G., De Melo M.L.M., Leão J.C., Carvalho A.T., Porter S., Duarte A.L.B.P., Dantas A.T., Gueiros L.A. (2019). Oral features of systemic sclerosis: A case–control study. Oral Dis..

[12] Holick M.F. (2008). The vitamin D deficiency pandemic and consequences for nonskeletal health: Mechanisms of action. Mol. Asp. Med..

[13] Cabras M., Gambino A., Broccoletti R., Lodi G., Arduino P.G. (2019). Treatment of angular cheilitis: A narrative review and authors’ clinical experience. Oral Dis..

[14] Hewison M. (2012). An update on vitamin D and human immunity. Clin. Endocrinol..

[15] Jamali N., Sorenson C.M., Sheibani N. (2018). Vitamin D and regulation of vascular cell function. Am. J. Physiol. Circ. Physiol..

[16] Al Hamad A., Lodi G., Porter S., Fedele S., Mercadante V. (2018). Interventions for dry mouth and hyposalivation in Sjögren’s syndrome: A systematic review and meta-analysis. Oral Dis..

[17] Rosen Y., Daich J., Soliman I., Brathwaite E., Shoenfeld Y. (2016). Vitamin D and autoimmunity. Scand. J. Rheumatol..

[18] Bi C.S., Wang J., Qu H.L., Li X., Tian B.M., Ge S., Chen F.M. (2019). Calcitriol suppresses lipopolysaccharide-induced alveolar bone damage in rats by regulating T helper cell subset polarization. J. Periodontal Res..

[19] Gong A., Chen J., Wu J., Li J., Wang L., Goltzman D., Miao D. (2018). 1,25-dihydroxyvitamin D deficiency accelerates alveolar bone loss independent of aging and extracellular calcium and phosphorus. J. Periodontol..

[20] Isola G., Alibrandi A., Rapisarda E., Matarese G., Williams R.C., Leonardi R. (2020). Association of vitamin D in patients with periodontitis: A cross-sectional study. J. Periodontal Res..

[21] Hu X., Niu L., Ma C., Huang Y., Yang X., Shi Y., Pan C., Liu J., Wang H., Li Q. (2019). Calcitriol decreases live Porphyromonas gingivalis internalized into epithelial cells and monocytes by promoting autophagy. J. Periodontol..

[22] Millen A.E., Hovey K.M., LaMonte M.J., Swanson M., Andrews C.A., Kluczynski M.A., Genco R.J., Wactawski-Wende J. (2013). Plasma 25-Hydroxyvitamin D Concentrations and Periodontal Disease in Postmenopausal Women. J. Periodontol..

[23] Dietrich T., Nunn M., Dawson-Hughes B., Bischoff-Ferrari H.A. (2005). Association between serum concentrations of 25-hydroxyvitamin D and gingival inflammation. Am. J. Clin. Nutr..

[24] Isola G., Lo Giudice A., Polizzi A., Alibrandi A., Murabito P., Indelicato F. (2021). Identification of the different salivary Interleukin-6 profiles in patients with periodontitis: A cross-sectional study. Arch. Oral Biol..

[25] Masi A.T. (1980). Subcommittee for Scleroderma Criteria of the American Rheumatism Association Diagnostic and Therapeutic Criteria Committee Preliminary criteria for the classification of systemic sclerosis (scleroderma). Arthritis Rheum..

[26] White B., Bauer E.A., Goldsmith L.A., Hochberg M.C., Katz L.M., Korn J.H., Lachenbruch P.A., LeRoy E.C., Mitrane M.P., Paulus H.E. (1995). Guidelines for clinical trials in systemic sclerosis (scleroderma). I. Disease-modifying interventions. The American College of Rheumatology Committee on Design and Outcomes in Clinical Trials in Systemic Sclerosis. Arthritis Rheum..

[27] Isola G., Polizzi A., Patini R., Ferlito S., Alibrandi A., Palazzo G. (2020). Association among serum and salivary A. actinomycetemcomitans specific immunoglobulin antibodies and periodontitis. BMC Oral Health.

[28] Antonoglou G.N., Suominen A.L., Knuuttila M., Ylöstalo P., Ojala M., Männistö S., Marniemi J., Lundqvist A., Tervonen T. (2015). Associations Between Serum 25-Hydroxyvitamin D and Periodontal Pocketing and Gingival Bleeding: Results of a Study in a Non-Smoking Population in Finland. J. Periodontol..

[29] Männistö S., Virtanen M., Mikkonen T., Pietinen P. (1996). Reproducibility and validity of a food frequency questionnaire in a case-control study on breast cancer. J. Clin. Epidemiol..

[30] Furst D.E., Clements P.J., Steen V.D., Medsger T.A., Masi A.T., D’Angelo W.A., Lachenbruch P.A., Grau R.G., Seibold J.R. (1998). The modified Rodnan skin score is an accurate reflection of skin biopsy thickness in systemic sclerosis. J. Rheumatol..

[31] O’Leary T.J., Drake R.B., Naylor J.E. (1972). The Plaque Control Record. J. Periodontol..

[32] Polizzi A., Santonocito S., Di Stefano M., Ferlito S., Indelicato F., Palazzo G. (2020). The effects on oral related quality of life induced by periodontitis in patients with juvenile idiopathic arthritis. Mediterr. J. Clin. Psychol..

[33] Zhang L., Duan Y., Zhang T.P., Huang X.L., Li B.Z., Ye D.Q., Wang J. (2017). Association between the serum level of vitamin D and systemic sclerosis in a Chinese population: A case control study. Int. J. Rheum. Dis..

[34] Ketharanathan V., Torgersen G.R., Petrovski B.É., Preus H.R. (2019). Radiographic alveolar bone level and levels of serum 25-OH-Vitamin D3 in ethnic Norwegian and Tamil periodontitis patients and their periodontally healthy controls. BMC Oral Heal..

[35] Arnson Y., Amital H., Agmon-Levin N., Alon D., Sánchez-Castañón M., López-Hoyos M., Matucci-Cerinic M., Szucs G., Shapira Y., Szekanecz Z. (2011). Serum 25-OH vitamin D concentrations are linked with various clinical aspects in patients with systemic sclerosis: A retrospective cohort study and review of the literature. Autoimmun. Rev..

[36] Lo Gullo A., Mandraffino G., Sardo M.A., D’Ascola A., Mamone F., Loddo S., Alibrandi A., Imbalzano E., Mormina E., Saitta C. (2013). Circulating progenitor cells in rheumatoid arthritis: Association with inflammation and oxidative stress. Scand. J. Rheumatol..

[37] Lo Gullo A., Mandraffino G., Bagnato G., Aragona C.O., Imbalzano E., D’Ascola A., Rotondo F., Cinquegrani A., Mormina E., Saitta C. (2015). Vitamin D Status in Rheumatoid Arthritis: Inflammation, Arterial Stiffness and Circulating Progenitor Cell Number. PLoS ONE.

[38] Zerr P., Vollath S., Palumbo-Zerr K., Tomcik M., Huang J., Distler A., Beyer C., Dees C., Gela K., Distler O. (2015). Vitamin D receptor regulates TGF-β signalling in systemic sclerosis. Ann. Rheum. Dis..

[39] Santonocito S., Ronsivalle V., Fichera G., Indelicato F. (2020). Psychological impact and patient perception of occlusion and orthodontic treatment in periodontitis patients. Mediterr. J. Clin. Psychol..

[40] Taylan A., Birlik M., Kenar G., Toprak B., Gundogdu B., Gurler O., Karakas B., Akıncı B., Sisman A.R. (2019). Osteoprotegrin interacts with biomarkers and cytokines that have roles in osteoporosis, skin fibrosis, and vasculopathy in systemic sclerosis: A potential multifaceted relationship between OPG/RANKL/TRAIL and Wnt inhibitors. Mod. Rheumatol..

[41] Yang C.-Y., Leung P.S.C., Adamopoulos I.E., Gershwin M.E. (2013). The Implication of Vitamin D and Autoimmunity: A Comprehensive Review. Clin. Rev. Allergy Immunol..

[42] Meghil M.M., Hutchens L., Raed A., Multani N.A., Rajendran M., Zhu H., Looney S., Elashiry M., Arce R.M., Peacock M.E. (2019). The influence of vitamin D supplementation on local and systemic inflammatory markers in periodontitis patients: A pilot study. Oral Dis..

[43] Chen Y.-C., Ninomiya T., Hosoya A., Hiraga T., Miyazawa H., Nakamura H. (2012). 1α,25-Dihydroxyvitamin D3 inhibits osteoblastic differentiation of mouse periodontal fibroblasts. Arch. Oral Biol..

[44] Agrawal D.K., Yin K. (2014). Vitamin D and inflammatory diseases. J. Inflamm. Res..

[45] Hiremath V.P., Rao C.B., Naik V., Prasad K.V. (2013). Anti-inflammatory effect of vitamin D on gingivitis: A dose-response randomised control trial. Oral Health Prev. Dent..

[46] Grenier D., Morin M., Fournier-Larente J., Chen H. (2016). Vitamin D inhibits the growth of and virulence factor gene expression by Porphyromonas gingivalis and blocks activation of the nuclear factor kappa B transcription factor in monocytes. J. Periodontal Res..

[47] Lai H., Fishman E.K., Gerstenblith G., Moore R., Brinker J.A., Keruly J.C., Chen S., Detrick B., Lai S. (2013). Vitamin D deficiency is associated with development of subclinical coronary artery disease in HIV-infected African American cocaine users with low Framingham-defined cardiovascular risk. Vasc. Health Risk Manag..

[48] Teles F.R., Teles R.P., Martin L., Socransky S.S., Haffajee A.D. (2012). Relationships Among Interleukin-6, Tumor Necrosis Factor-α, Adipokines, Vitamin D, and Chronic Periodontitis. J. Periodontol..

[49] Zhan Y., Samietz S., Holtfreter B., Hannemann A., Meisel P., Nauck M., Völzke H., Wallaschofski H., Dietrich T., Kocher T. (2014). Prospective Study of Serum 25-hydroxy Vitamin D and Tooth Loss. J. Dent. Res..

[50] Garcia M.N., Hildebolt C.F., Miley D.D., Dixon D.A., Couture R.A., Spearie C.L.A., Langenwalter E.M., Shannon W.D., Deych E., Mueller C. (2011). One-Year Effects of Vitamin D and Calcium Supplementation on Chronic Periodontitis. J. Periodontol..

[51] Miley D.D., Garcia M.N., Hildebolt C.F., Shannon W.D., Couture R.A., Spearie C.L.A., Dixon D.A., Langenwalter E.M., Mueller C., Civitelli R. (2009). Cross-Sectional Study of Vitamin D and Calcium Supplementation Effects on Chronic Periodontitis. J. Periodontol..

[52] Humphrey L.L., Fu R., Buckley D.I., Freeman M., Helfand M. (2008). Periodontal Disease and Coronary Heart Disease Incidence: A Systematic Review and Meta-analysis. J. Gen. Intern. Med..

[53] Amaliya A., Laine M.L., Delanghe J.R., Loos B.G., Van Wijk A.J., Van Der Velden U. (2015). Java project on periodontal diseases: Periodontal bone loss in relation to environmental and systemic conditions. J. Clin. Periodontol..

[54] Pink C., Kocher T.D., Meisel P., Dörr M., Markus M.R.P., Jablonowski L., Grotevendt A., Nauck M., Holtfreter B. (2015). Longitudinal effects of systemic inflammation markers on periodontitis. J. Clin. Periodontol..

[55] Polizzi A., Santonocito S., Vaccaro M., Fichera G., Torrisi S., Ronsivalle V., Palazzo G., Sicari F., Indelicato F. (2020). Relationship between periodontitis and psychosocial impact in patients with systemic sclerosis: A clinical study. Mediterr. J. Clin. Psychol..

[56] Bilgin Çetin M., Önder C., Orhan K., Kumbasar D., Serdar M.A., Ünsal E. (2020). Relationship of periodontitis and edentulism to angiographically diagnosed coronary artery disease: A cross-sectional study. J. Periodontal Res..

[57] Desai C.S., Lee D.C., Shah S.J. (2011). Systemic sclerosis and the heart: Current diagnosis and management. Curr. Opin. Rheumatol..

